# Supplementary material for: Resolving the full spectrum of human genome variation using Linked-Reads
Source: Genome Res. 2019 Apr;29(4):635–45. doi: 10.1101/gr.234443.118 (PMC6442396; doi:10.1101/gr.234443.118)
Supplement: Supplemental Material [file supp_gr.234443.118_Supplemental_longranger-2.2.zip › longranger-2.2.tar.bz2!/lr2.2-public/lib/stan/cmdstan/stan/doc/api/html/namespacemembers_r.html]

Stan: Namespace Members


|  |  |
| --- | --- |
|  | Stan  2.5.0  probability, sampling & optimization |


- Main Page
- Related Pages
- Namespaces
- Classes
- Files

- Namespace List
- Namespace Members

- All
- Functions
- Variables
- Typedefs
- Enumerations
- Enumerator

- a
- b
- c
- d
- e
- f
- g
- h
- i
- j
- l
- m
- n
- o
- p
- q
- r
- s
- t
- u
- v
- w


All Classes Namespaces Files Functions Variables Typedefs Enumerations Enumerator Friends Macros Pages

Here is a list of all namespace members with links to the namespace documentation for each member:

### - r -

- rank()
  : stan::math
- rayleigh\_ccdf\_log()
  : stan::prob
- rayleigh\_cdf()
  : stan::prob
- rayleigh\_cdf\_log()
  : stan::prob
- rayleigh\_log()
  : stan::prob
- rayleigh\_rng()
  : stan::prob
- read\_corr\_L()
  : stan::prob
- read\_corr\_matrix()
  : stan::prob
- read\_cov\_L()
  : stan::prob
- read\_cov\_matrix()
  : stan::prob
- real\_argument
  : stan::gm
- recover\_memory()
  : stan::agrad
- recover\_memory\_nested()
  : stan::agrad
- remove\_loop\_identifier\_f
  : stan::gm
- rep\_array()
  : stan::math
- rep\_matrix()
  : stan::math
- rep\_row\_vector()
  : stan::math
- rep\_vector()
  : stan::math
- resize()
  : stan::math
- returns\_type()
  : stan::gm
- rising\_factorial()
  : stan::agrad
  , stan::math
- round()
  : stan::agrad
- row()
  : stan::math
- row\_vector\_d
  : stan::math
- row\_vector\_fd
  : stan::agrad
- row\_vector\_ffd
  : stan::agrad
- row\_vector\_ffv
  : stan::agrad
- row\_vector\_fv
  : stan::agrad
- ROW\_VECTOR\_T
  : stan::gm
- row\_vector\_v
  : stan::agrad
- rows()
  : stan::math
- rows\_dot\_product()
  : stan::agrad
  , stan::math
- rows\_dot\_self()
  : stan::agrad
  , stan::math
- run\_markov\_chain()
  : stan::common


---

[ Stan Home Page ]

*© 2011–2014,
Stan Development Team.*
